# Health-related quality of life of postmenopausal Chinese women with hormone receptor-positive early breast cancer during treatment with adjuvant aromatase inhibitors: a prospective, multicenter, non-interventional study

**DOI:** 10.1186/s12955-016-0446-2

**Published:** 2016-03-24

**Authors:** Ayong Cao, Jin Zhang, Xiaoan Liu, Weizhu Wu, Yinhua Liu, Zhimin Fan, Anqin Zhang, Tianning Zhou, Peifen Fu, Shu Wang, Quchang Ouyang, Jinhai Tang, Hongchuan Jiang, Xiaohua Zhang, Da Pang, Jianjun He, Linxiang Shi, Xianming Wang, Yuan Sheng, Dahua Mao, Zhimin Shao

**Affiliations:** Department of Breast Surgery, Fudan University Shanghai Cancer Center, No. 270 Dongan Road, Xuhui District, Shanghai, 200032 China; Department of Breast Cancer, Tianjin Medical University Cancer Institute and Hospital, Tianjin, 300060 China; Department of Breast Surgery, Jiangsu Province Hospital, Nanjing, China; Department of Oncology Surgery, Ningbo Medical Treatment Center Lihuili Hospital, Ningbo, 315000 China; Breast Disease Center, Peking University First Hospital, Beijing, 100034 China; Department of Breast Surgery, First Hospital of Jilin University, Changchun, 130021 China; Breast Disease Center, Guangdong Women and Children’s Hospital and Health Institute, Guangzhou, 510010 China; Department of Breast Disease, Yunnan Tumor Hospital, Kunming, 650118 China; Breast Disease Center, First Affiliated Hospital of Medicine College of Zhejiang University, Hangzhou, 310006 China; Breast Disease Center, Peking University People’s Hospital, Beijing, 100044 China; Medical Oncology Center, Hunan Tumor Hospital, Changsha, 410000 China; Department of General Surgery, Jiangsu Cancer Hospital, Nanjing, 210009 China; Department of General Surgery, Beijing Chao-Yang Hospital, Capital Medical University, Beijing, 100020 China; Department of Oncology Surgery, The First Affiliated Hospital of Wenzhou Medical College, Wenzhou, 325000 China; Department of Breast Surgery, The Tumor Hospital of Harbin Medical University, Harbin, 150040 China; Department of Surgical Oncology, The First Affiliated Hospital, School of Medicine, Xi’an Jiaotong University, Xi’an, 710000 China; Department of Breast and Thyroid Surgery, Shanghai Tenth People’s Hospital, Shanghai, 200072 China; Department of Breast and Thyroid Surgery, Shenzhen Second Hospital, Shenzhen, 518000 China; Department of Breast Surgery, Shanghai Changhai Hospital, Shanghai, 200433 China; Department of Breast Surgery, The Affiliated Hospital of Guiyang Medical College, Guiyang, Guizhou 550004 China

**Keywords:** Quality of life, Prospective, Functional Assessment of Cancer Therapy-Breast, Trial outcome index, Hormone receptor-positive early-stage breast cancer postmenopause, Adjuvant, Aromatase inhibitors, Early breast cancer

## Abstract

**Background:**

Estimating quality of life (QoL) in patients with breast cancer is of importance in assessing treatment outcomes. Adjuvant endocrine therapy is widely used for hormone receptor-positive (HR+) early-stage breast cancer (EBC), and evidence suggests that aromatase inhibitors (AIs) may improve QoL for these patients. This study evaluated QoL in postmenopausal Chinese patients with HR+ EBC taking AIs.

**Methods:**

This was a prospective, multicenter, and observational study that had no intent to intervene in the current treatment of recruited patients. Eligible patients were recruited within 7 days of beginning adjuvant treatment with AIs. The Functional Assessment of Cancer Therapy-Breast (FACT-B) scale was used to evaluate the patients’ QoL. Data were collected at baseline and at 6, 12, 18, and 24 months.

**Results:**

From June 2010 to October 2013, a total of 494 patients with HR+ EBC were recruited from 21 centers. There was a 7.51-point increase in the patients’ mean FACT-B trial outcome index (TOI), from 90.69 at baseline to 98.72 at 24 months (*P* < .0001). The mean TOI scores at baseline, 6, 12, and 18 months were 90.69, 94.36, 97.71, and 96.75, respectively (*P* < .0001, for all). The mean (FACT-B) emotional well-being subscale scores at baseline, 6, 12, 18, and 24 months were 16.32, 16.55, 17.34 (*P* < .0001), 17.47 (*P* < .0001), and 17.85 (*P* < .0001), respectively, and social well-being scores were 18.61, 19.14 (*P* < .04), 19.35 (*P* < .008), 18.32, and 18.40, respectively. In the mixed model, baseline TOI, clinical visits, prior chemotherapies, age group, and axillary lymph-node dissection presented statistically significant effects on the change of FACT-B TOI and FACT-B SWB, whereas only baseline TOI, clinical visits, and prior chemotherapies presented statistically significant effects on the change of FACT-B EWB. FACT-B TOI, being the most pertinent and precise indicator of patient-reported QoL, demonstrated significant changes reflecting clinical benefit of adjuvant AIs endocrine therapy in the QoL of HR + EBC patients.

**Conclusions:**

The study demonstrated significant improvements in the long-term QoL of postmenopausal Chinese patients with HR+ EBC at 6, 12, 18, and 24 months after starting treatment with AIs. The current study indicates improved long-term QoL with AI adjuvant treatment, which will aid clinicians in optimizing treatment to yield effective healthcare outcomes.

**Trial registration:**

Clinicaltrials.gov NCT01144572

## Background

Breast cancer is the most common cancer in women worldwide, with nearly 1.7 million new cases diagnosed in 2012, representing about 12 % of all new cancer cases and 25 % of all cancers in women [1]. China accounts for nearly 12.2 % of all newly diagnosed breast cancer cases and about 9.6 % of all deaths from breast cancer worldwide [2]. In the past two decades, although the incidence of breast cancer has steadily increased, the death rate from the disease has declined [3]. Moreover, awareness of patients’ quality of life (QoL) during treatment is increasing, making it important for physicians and patients to consider therapeutic efficacy, safety of medical interventions, and QoL when selecting a treatment program. In addition, health-related QoL (HRQoL) is now being considered as an important end point in cancer clinical trials, which could contribute to improved treatment outcomes [4, 5].

Because various studies have consistently demonstrated superior efficacy and safety of the third-generation aromatase inhibitors (AIs) compared with those of tamoxifen alone in postmenopausal women with ER (+) breast cancer [6, 7], AIs are widely administered as standard adjuvant endocrine therapy in this population [8]. Previous researches suggested that treatment of patients with early-stage breast cancer (EBC) with third-generation AIs (e.g., anastrozole, letrozole, and exemestane) may improve their QoL [6–9]. However, this therapy is associated with persistent side effects and toxicity, which lead to negatively impact the patient’s QoL [10].

The Functional Assessment of Cancer Therapy-Breast (FACT-B), developed by Rush-Presbyterian-St. Luke’s Medical Center, Chicago, United States, is a widely used international scale to assess the QoL of patients with cancer [11, 12]. The use of such internationally consistent and validated scales to evaluate the QoL of Chinese patients will help improve the understanding of breast cancer outcomes. Specifically, they should enable physicians and patients to more clearly understand the long-term effects of AI therapy in breast cancer while making important treatment decisions. To date no nationwide, prospective, multicenter study has been available in China to evaluate the QoL of postmenopausal patients with early-stage breast cancer (EBC) positive (+) for hormone receptors (HR) treated with adjuvant therapy with AIs. It is of vital importance to evaluate the quality of life of Chinese patients using an internationally accepted method, so that the Chinese physicians are better knowledgeable about the effects of AIs on the long-term therapy when making therapeutic decisions. Because QoL studies can further indicate the directions needed for efficient treatment of cancer, the current study was conducted to evaluate the QoL of postmenopausal patients with HR (+) EBC receiving adjuvant treatment with AIs [13, 14].

## Methods

### Study design

This was the first prospective, multicenter, observational, non-interventional study in Chinese postmenopausal women [HR (+) and EBC] undergoing adjuvant treatment with AIs. There was no intent to intervene in the current treatment of the recruited patients (NCT01144572).

### Participants

The following were the screening criteria: postmenopausal women, age ≤70 years, histologically documented HR (+) EBC, currently on adjuvant endocrine therapy with AIs and indications approved by the State Food and Drug Administration (SFDA), or AI adjuvant therapy started within the past 7 days. Women were eligible to be included in the study if they had simple mastectomy, breast-conserving surgery, or axillary lymph node dissection or sampling. Patients were excluded from the analysis if they were disinterested in participating, unable to adhere to the trial requirements for any reason, or were using AIs not approved by the SFDA for initial adjuvant endocrine therapy for EBC. Written informed consent for the data collection was obtained at the clinic visit after eligibility requirements were confirmed by the investigator. At any point during the study, patients could discontinue from the final analysis if it was their own decision, they had discontinued previous AI medication, or they had a documented evidence of disease relapse or progression.

The study was performed in accordance with Declaration of Helsinki and Good Clinical Practice Guidelines, with approval obtained from each center’s independent ethics committee.

### Study objective

The primary objective was to evaluate overall QoL in postmenopausal patients with HR(+) EBC during adjuvant treatment with AIs by assessing the change in the trial outcome index (TOI) of the FACT-B questionnaire from baseline to 24 months. The secondary objective was to evaluate the QoL in the same population during adjuvant treatment with AIs by examining FACT-B questionnaire results at several time points (baseline, 6, 12, and 18 months) on three FACT-B measures: TOI, emotional well-being (EWB) subscale scores, and social well-being (SWB) subscale scores. In addition, the factors affecting the QoL of patients with breast cancer were also determined.

### Data collection

Data for demography and pathological examination for breast cancer were obtained for all the patients included in the analysis. Demographic parameters recorded were age, ethnic background, height, weight, basal metabolic index, and World Health Organization (WHO) performance status score. Pathological examination for breast cancer included assessment of the primary site, histological type, histological grading, and TNM stage. In addition, data were recorded for status of estrogen and progesterone receptors, history of breast cancer surgery, history of prior chemotherapy or radiotherapy, and use of AIs.

### Quality of life assessment

QoL was assessed using the FACT-B questionnaire from baseline to 6, 12, 18, and 24 months. The TOI (sum of the physical well-being, functional well-being, and breast cancer–specific questions in the FACT-B) was regarded as the primary end point. An increase in TOI of more than 5 points from baseline, disease progression, or death was considered a clinically meaningful improvement in QoL. FACT-B instrument uses a 5-point rating scale, including measures for physical, social/family, emotional, and functional well-being. The instrument was used to inquire respondents on how true each statement was for the past 7 days and rate them. Response scales ranged from 0 (not at all) to 4 (very much). A one-on-one discussion was scheduled to collect data on demographic characteristics and QoL.

### Statistical methods

The sample size was not formally calculated as this was a cohort study and there was no formal comparison between the groups. It was projected that 500 patients would be enrolled within 3 years given the patient pool capacity in the participating sites. The primary QoL analysis, inclusive of all available questionnaires, was based on the patients who completed the informed consent form (ICF) and had one clinical visit included in the final analysis. The analysis was performed using the MIXED procedure of the SAS statistical software (SAS Institute, Cary, North Carolina, USA), which has the advantage of handling missing data without any imputation of missing values, provided that data are missing at random. Baseline covariates such as TOI score, prior chemotherapy (yes/no), age group, mastectomy (yes/no), and axillary clearance (yes/no) were included in the model regardless of statistical significance. This repeated measures analysis also included time from baseline, as both fixed and random polynomial effects, to allow patient-specific time trends and intercepts (described via random effects) to vary around an overall average trend and intercept (described via the fixed effects). The results of the analyses are presented in terms of adjusted means, associated confidence intervals (CIs), and *P* values. The mean score change from baseline was calculated for each patient with valid baseline questionnaires.

## Results

### Patient disposition

**Fig. 1:**
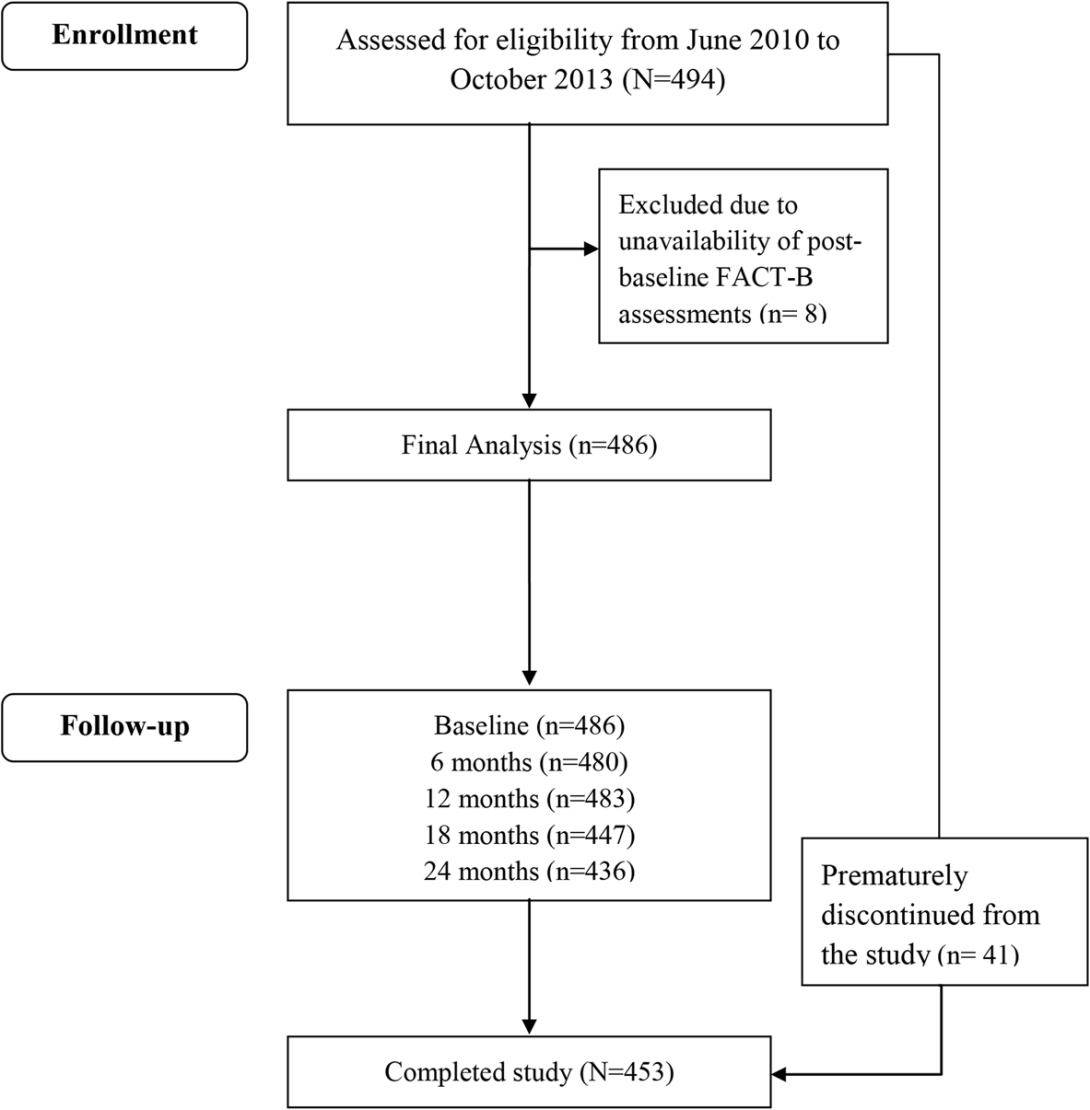
Study flow chart

From June 2010 to October 2013, a total of 494 patients were enrolled in the study; of these patients, 453 (91.7 %) completed the study and 41 (8.3 %) prematurely discontinued from the study as a result of loss to follow-up (5.1 %), disease relapse/progression (1.2 %), failure to adhere to inclusion/exclusion criteria (0.6 %), voluntarily discontinuation (0.4 %), cessation of AI therapy (0.4 %) and other (0.6 %). Thirty-one patients had at least one protocol violation, majorly because of failure to adhere to inclusion criteria pertaining to postmenopausal women aged ≤70 years (*n* = 21), starting of AI adjuvant therapy within the past 7 days (*n* = 7), and unavailability of FACT-B data for clinical visits 2–5 (*n* = 8). Subsequently, a total of 486 patients (98.4 %) were included for the final analysis (Fig. 1).

### Demographics and other baseline characteristics

All the 486 patients were of Chinese ethnic origin with a mean age of 57.3 years (range: 27–79 years), including 316 (65 %) women aged ≤60 years and 170 (35 %) women aged >60 years. Other baseline characteristics are listed in Table 1. Most patients had unilateral breast cancer, except three patients who reported bilateral primary breast cancer. More than half (372; 76.5 %) of the patients had estrogen receptor (ER, +)/progesterone receptor (PR, +) breast cancer, 99 (20.4 %) patients had ER(+)/PR(−) breast cancer, and 15 (3.1 %) patients had ER(−)/PR(+) breast cancer.

**Table 1:** Baseline characteristics

Parameter		(*N* = 486)
Weight (kg)	N (missing)	486 (0)
Mean (SD)		59.8 (8.93)
Median		59.0
Min ~ max		35,105
Height (cm)	N(missing)	486 (0)
Mean (SD)		159.4 (4.58)
Median		160.0
Min ~ max		143,175
BMI (kg/m^2^)	N(missing)	486 (0)
Mean (SD)		23.5 (3.25)
Median		23.0
Min ~ max		16,39
WHO Performance Status Score	Activities limited	148 (30.5 %)
	Normal activities	329 (67.7 %)
	Bedridden time ≤ 50 %	9 (1.9 %)
	Total	486 (100.0 %)
Primary site	Right breast	240 (49.4 %)
	Left breast	243 (50.0 %)
	Left breast, right breast	3 (0.6 %)
	Total	486 (100.0 %)
Histological type	Invasive carcinoma	463 (95.3 %)
	Carcinoma in-situ	23 (4.7 %)
	Total	486 (100.0 %)
Histological grading	Unevaluable(GX)	58 (11.9 %)
	Poorly differentialted(G3)	84 (17.3 %)
	Well differentiated(G1)	56 (11.5 %)
	Undifferentiated(G4)	9 (1.9 %)
	Moderately differentiated(G2)	279 (57.4 %)
	Total	486 (100.0 %)
ER status	Positive	471 (96.9 %)
	Negative	15 (3.1 %)
	Total	486 (100.0 %)
PR status	Positive	387 (79.6 %)
	Negative	99 (20.4 %)
	Total	486 (100.0 %)
ER and PR status	ER positive,PR positive	372 (76.5 %)
	ER positive,PR negative	99 (20.4 %)
	ER negative,PR positive	15 (3.1 %)
	Total	486 (100.0 %)
Mastectomy	No	36 (7.4 %)
	Yes	450 (92.6 %)
	Total	486 (100.0 %)
Breast-conserving surgery	No	450 (92.6 %)
	Yes	36 (7.4 %)
	Total	486 (100.0 %)
Axillary lymph node dissection	No	103 (21.2 %)
	Yes	383 (78.8 %)
	Total	486 (100.0 %)
Axillary lymph node sampling	No	312 (64.2 %)
	Yes	174 (35.8 %)
	Total	486 (100.0 %)
Prior chemotherapy	No	227 (46.7 %)
	Yes	259 (53.3 %)
	Total	486 (100.0 %)
Prior radiotherapy	No	432 (88.9 %)
	Yes	54 (11.1 %)
	Total	486 (100.0 %)

A total of 450 (92.6 %) patients had mastectomy, 36 (7.4 %) had breast-conserving surgery, 383 (78.8 %) had axillary lymph-node dissection, and 312 (64.2 %) had undergone axillary lymph-node sampling. Fifteen patients had an unknown AI use status; 426 patients were taking anastrozole, with 416 (97.7 %) treated for more than 2 years. The detailed information is given in Table 2. Among the 486 patients, only 1 patient reported hormone replacement therapy, 152 (31.3 %) patients were found to have major medical conditions other than breast cancer, and 92 (18.9 %) patients had major surgical therapies other than breast cancer surgery.

**Table 2:** Use of aromatase inhibitors (final analysis set)

		Final analysis set
	Treatment duration (months)	(*N* = 486) *n* (%)
Anastrozole	<6	1 (0.2)
	6–12	5 (1.2)
	12–18	1 (0.2)
	18–24	3 (0.7)
	>24	416 (97.7)
	Total	426 (100)
	Missing	15
Anastrozole/exemestane	>24	1 (100)
	Total	1 (100)
Anastrozole/letrozole	>24	5 (100)
	Total	5 (100)
Exemestane	>24	10 (100)
	Total	10 (100)
Letrozole	6–12	2 (6.9)
	>24	27 (93.1)
	Total	29 (100)

### Primary endpoint-change in the FACT-B TOI from baseline to 24 months

A significant improvement was observed on the mean FACT-B TOI from baseline (mean: 90.69) to 24 months (mean: 98.72; *P* < .0001) (Table 3). In the MIXED model, baseline TOI, visit, prior chemotherapies, age group, and axillary lymph-node dissection presented statistically significant effects on the change of FACT-B TOI; however, mastectomy presented no statistically significant effects on the change of FACT-B TOI (Table 4). Lower boundary of the CI of the least squares (LS) mean (adjusted mean) change from baseline to 24 months in FACT-B TOI was >0, indicative of an increased TOI at 24 months of treatment. Moreover, LS mean change from 6 to 24 months of treatment was −4.48 (*P* < .0001). The LS mean of changes from baseline in FACT-B TOI with 95 % CIs is given in Table 4.

**Table 3:** FACT-B TOI at each post-treatment visit and changes from baseline (final analysis set)

		Final analysis set (*N* = 486)	
Visit	Clinical efficacy	TOI	TOI change from baseline
Baseline	N (missing)	486 (0)	
	Mean (SD)	90.69 (19.526)	
	Median	89.65	
	Min–max	31.3–137.0	
6 months	N (missing)	480 (6)	480 (6)
	Mean (SD)	94.36 (18.995)	3.58 (15.312)
	Median	94.00	1.50
	Min–max	58.5–141.0	−61.0 to 61.8
	*P* value in comparison to baseline		<.0001
12 months	N (missing)	483 (3)	483 (3)
	Mean (SD)	97.71 (20.670)	7.03 (19.241)
	Median	98.00	3.83
	Min–max	47.0–140.0	−50.0 to 82.0
	*P* value in comparison to baseline		<.0001
18 months	N (missing)	447 (39)	447 (39)
	Mean (SD)	96.75 (22.074)	5.18 (21.378)
	Median	95.83	4.00
	Min–max	54.0–142.0	−58.0 to 78.0
	*P* value in comparison to baseline		<.0001
24 months	N (missing)	436 (50)	436 (50)
	Mean (SD)	98.72 (22.672)	7.51 (22.476)
	Median	97.58	6.00
	Min–max	49.0–140.0	−59.0 to 81.3
	*P* value in comparison to baseline		<.0001

*FACT-B* Functional Assessment of Cancer Therapy-Breast, *SD* standard deviation, *TOI* trial outcome index

**Table 4:** LS mean of changes from baseline in FACT-B TOI with 95 % CIs (final analysis set)

		Final analysis set (*N* = 486)		
		LS Mean	95 % CI	*P* value
Visit	6 months	4.04	1.64–6.43	–
	12 months	7.10	4.57–9.63	–
	18 months	6.54	3.84–9.25	–
	24 months	8.51	5.75–11.27	–
	6–12 months	−3.06	−4.06 to −2.06	<.0001
	6–18 months	−2.50	−3.83 to −1.17	.0002
	6–24 months	−4.48	−5.96 to −2.99	<.0001
	12–18 months	0.56	−0.59 to 1.70	.3405
	12–24 months	−1.41	−2.78 to −0.05	.0422
	18–24 months	−1.97	−2.92 to −1.02	<.0001
Prior chemotherapies	Yes	10.99	8.20–13.77	–
	No	2.11	−0.69 to 4.90	–
	Yes–No	8.88	6.36–11.40	<.0001
Age group	>60 years	8.04	5.04–11.05	–
	≤60 years	5.05	2.44–7.66	–
	>60 to ≤60 years	2.99	0.37–5.61	.0256
Mastectomy	Yes	7.63	5.88–9.38	–
	No	5.46	0.90–10.03	–
	Yes–No	2.17	−2.63 to 6.97	.3752
Axillary lymph-node dissection	Yes	4.77	2.00–7.55	–
	No	8.32	5.22–11.43	–
	Yes–No	−3.55	−6.68 to −0.41	.0268

*CI* confidence interval, *FACT-B* Functional Assessment of Cancer Therapy-Breast, *LS* least squares, *TOI* trial outcome index

### Secondary end points

#### Changes in the FACT-B TOI from baseline to 6, 12, and 18 months

The mean FACT-B TOI was 90.69, 94.36, 97.71, and 96.75 at baseline, 6, 12, and 18 months, respectively, contributing to the increases from baseline 3.58, 7.03, and 5.18 at 6, 12, and 18 months, respectively (Table 3). The mean change in FACT-B TOI was significant at all time points as compared with baseline (*P* < .0001, for all).

#### Changes in the FACT-B EWB from baseline to 6, 12, 18, and 24 months

The mean FACT-B EWB was 16.32, 16.55, 17.34, 17.47, and 17.85 at baseline, 6, 12, 18, and 24 months, respectively, contributing to the mean increases from baseline 0.18, 1.03, 0.90, and 1.36 at 6, 12, 18, and 24 months, respectively (Table 5). The mean change in FACT-B EWB was significant at 12, 18, and 24 months as compared with baseline (*P* < .0001, for all).

**Table 5:** FACT-B EWB scores at each post-treatment visit and changes from baseline (final analysis set)

		Final analysis set (*N* = 486)	
Visit	Clinical efficacy	Total score	Change from baseline in total score
Baseline	N (missing)	486 (0)	
	Mean (SD)	16.32 (4.497)	
	Median	17.00	
	Min–max	2.0–24.0	
6 months	N (missing)	480 (6)	480 (6)
	Mean (SD)	16.55 (4.318)	0.18 (3.741)
	Median	17.00	0.00
	Min–max	6.0–24.0	−13.0 to 12.0
	*P* value in comparison to baseline		0.2846
12 months	N (missing)	483 (3)	483 (3)
	Mean (SD)	17.34 (4.083)	1.03 (4.314)
	Median	17.00	1.00
	Min–max	6.0–24.0	−11.0 to 17.0
	*P* value in comparison to baseline		<.0001
18 months	N (missing)	447 (39)	447 (39)
	Mean (SD)	17.47 (4.215)	0.90 (4.600)
	Median	18.00	1.00
	Min–max	0.0–24.0	−12.0 to 17.0
	*P* value in comparison to baseline		<.0001
24 months	N (missing)	436 (50)	436 (50)
	Mean (SD)	17.85 (4.163)	1.36 (5.027)
	Median	18.00	1.00
	Min–max	0.0–24.0	−14.0 to 16.0
	*P* value in comparison to baseline		<.0001

*EWB* emotional well-being, *FACT-B* Functional Assessment of Cancer Therapy-Breast, *SD* standard deviation

In the MIXED model, baseline TOI, clinical visit, and prior chemotherapies had statistically significant effects on the change in FACT-B TOI, whereas age group, mastectomy, and axillary lymph-node dissection had no statistically significant effects on the change in FACT-B EWB (Table 6). In the MIXED model including baseline TOI, clinical visit, prior chemotherapies, age group, mastectomy, and axillary lymph-node dissection, the lower boundaries of the CIs of the LS mean (adjusted mean) changes from baseline to 12, 18, and 24 months in FACT-B EWB were all >0, indicating increased scores at 12, 18, and 24 months of treatment. The changes from 6 months of treatment to 12, 18, and 24 months of treatment are detailed in Table 6.

**Table 6:** LS mean of changes from baseline in FACT-B EWB score with 95 % CIs (final analysis set)

		Final analysis set (*N* = 486)		
		LS mean	95 % CI	*P* value
Follow-up	6 months	−0.00	−0.53 to 0.53	–
	12 months	0.76	0.22–1.30	–
	18 months	0.92	0.36–1.48	–
	24 months	1.29	0.71–1.86	–
	6–12 months	−0.76	−1.04 to −0.48	<.0001
	6–18 months	−0.92	−1.26 to −0.58	<.0001
	6–24 months	−1.29	−1.65 to −0.93	<.0001
	12–18 months	−0.16	−0.46 to 0.14	.2895
	12–24 months	−0.53	−0.88 to −0.18	.0033
	18–24 months	−0.37	−0.67 to −0.07	.0172
Prior chemotherapies	Yes	1.54	0.96–2.12	–
	No	−0.06	−0.64 to 0.53	–
	Yes–No	1.60	1.05–2.14	<.0001
Age group	>60 years	1.01	0.38–1.64	–
	≤60 years	0.47	−0.07–1.01	–
	>60 years – ≤60 years	0.54	−0.03 to 1.10	.0613
Mastectomy	Yes	1.20	0.85–1.55	–
	No	0.29	−0.68 to 1.25	–
	Yes–No	0.91	−0.12 to 1.94	.0823
Axillary lymph-node dissection	Yes	0.48	−0.10 to 1.06	–
	No	1.01	0.36–1.65	–
	Yes–No	−0.53	−1.20–0.14	.1233

*CI* confidence interval, *EWB* emotional well-being, *FACT-B* Functional Assessment of Cancer Therapy-Breast, *LS* least squares

#### Changes in the FACT-B SWB from baseline to 6, 12, 18, and 24 months

The mean FACT-B SWB was 18.61, 19.14, 19.35, 18.32, and 18.40 at baseline, 6, 12, 18, and 24 months, respectively, contributing to the mean changes from baseline 0.54, 0.75, −0.45, and −0.19 at 6, 12, 18, and 24 months, respectively. The mean change in FACT-B SWB was significant at 6 months (*P* < .0435) and 12 months (*P* < .0086) as compared with the baseline value (Table 7). In the MIXED model, baseline TOI, clinical visit, prior chemotherapies, age group, and axillary lymph-node dissection presented statistically significant effects on the FACT-B SWB, whereas mastectomy presented no statistically significant effects on the FACT-B SWB. The LS means of changes from baseline in the FACT-B SWB score with 95 % CI are presented in Table 8.

**Table 7:** FACT-B SWB scores at each post-treatment visit and changes from baseline (final analysis set)

		Final analysis set (*N* = 486)	
Visit	Clinical efficacy	Total score	Change from baseline in total score
Baseline	N (missing)	486 (0)	
	Mean (SD)	18.61 (6.732)	
	Median	19.30	
	Min–max	0.0–28.0	
6 months	N (missing)	480 (6)	480 (6)
	Mean (SD)	19.14 (6.068)	0.54 (5.872)
	Median	19.83	0.00
	Min–max	0.0–28.0	−24.5 to 28.0
	*P* value in comparison to baseline		.0435
12 months	N (missing)	483 (3)	483 (3)
	Mean (SD)	19.35 (6.531)	0.75 (6.245)
	Median	20.00	0.00
	Min–max	0.0–28.0	−21.3 to 28.0
	*P* value in comparison to baseline		.0086
18 months	N (missing)	447 (39)	447 (39)
	Mean (SD)	18.32 (6.900)	−0.45 (6.605)
	Median	19.00	0.00
	Min–max	0.0–28.0	−20.0 to 28.0
	*P* value in comparison to baseline		0.1523
24 months	N (missing)	436 (50)	436 (50)
	Mean (SD)	18.40 (7.422)	−0.19 (6.961)
	Median	19.83	0.00
	Min–max	1.0–28.0	−21.0 to 28.0
	*P* value in comparison to baseline		0.5677

*FACT-B* Functional Assessment of Cancer Therapy-Breast, *SD* standard deviation, *SWB* social well-being

**Table 8:** LS mean of changes from baseline in FACT-B SWB score with 95 % CIs (final analysis set)

		Final analysis set (*N* = 486)		
		LS mean	95 % CI	*P* value
Follow-up	6 months	0.85	0.07–1.63	–
	12 months	1.02	0.23–1.80	–
	18 months	0.13	−0.69 to 0.95	–
	24 months	0.22	−0.62 to 1.07	–
	6–12 months	−0.16	−0.63 to 0.30	.4849
	6–18 months	0.72	0.21–1.23	.0057
	6– 24 months	0.63	0.05–1.20	.0323
	12–18 months	0.89	0.49–1.28	<.0001
	12–24 months	0.79	0.27–1.31	.0029
	18–24 months	−0.09	−0.51 to 0.32	.6622
Prior chemotherapies	Yes	1.75	0.90–2.61	-
	No	−0.64	−1.49 to 0.21	-
	Yes–No	2.40	1.60-3.19	<.0001
Age group	>60 years	1.30	0.39–2.22	–
	≤60 years	−0.19	−0.99 to 0.60	–
	>60 to ≤60 years	1.50	0.68–2.32	.0004
Mastectomy	Yes	0.72	0.21–1.24	–
	No	0.39	−1.02 to 1.79	–
	Yes–No	0.33	−1.16 to 1.83	.6596
Axillary lymph-node dissection	Yes	0.06	−0.78 to 0.90	–
	No	1.05	0.10–2.00	–
	Yes–No	−0.99	−1.97 to −0.01	.0482

## Discussion

This first prospective, multicenter, observational, and non-interventional study in Chinese postmenopausal women with HR (+) breast cancer or HR+ EBC undergoing adjuvant treatment with AIs demonstrated significant improvements in the long-term QoL when evaluated at 6, 12, 18, and 24 months post-AI treatment. The study will provide insight to the concerns of the survivors and convey the information to clinical decision-makers who can use it to create patient-centered solutions.

Shen et al. [15] conducted a clinical controlled study in 522 patients to investigate the QoL of Chinese women with breast cancer by using the FACT-B scale. Regression analysis demonstrated that there was a significant increase in FACT-B TOI of the patients >50 years of age, with low-stage cancer, and with high education and income. This was a cross-sectional, single-center observational study in Chinese patients that provided valuable information about factors affecting the QoL of Chinese women with breast cancer. However, dynamic changes on the long-term QoL were not assessed.

Mixed results have been obtained from previous studies conducted on the HRQoL outcomes following AI therapy. Some studies have suggested that treatment with AIs may improve QoL, whereas others have indicated no increased benefit in HRQoL with AI compared with tamoxifen [16]. Fallowfield et al. first published results from a longitudinal follow-up of the impact of 5 years of adjuvant AI (anastrozole) and tamoxifen (alone/combination) treatment on HRQoL in a subgroup of 1105 patients (ATAC) [12]. Improvement in TOI was comparable in both the treatment groups over the 5-year recommended adjuvant treatment period [11, 12].

A 5-point increase in the FACT-B TOI is considered as a significant clinical benefit [17, 18]. The results of our current analysis are in line with those of the above-mentioned studies and demonstrated a significant increase of more than 5 points from baseline (mean: 90.69) to 24 months (mean: 98.72; *P* < .0001), thereby indicating significant clinical benefit of adjuvant AIs endocrine therapy in the QoL of patients with breast cancer.

Kwan et al. [19] demonstrated that the improvement of FACT-B TOI score in elderly patients (>60 years of age) was better than that in young patients. In addition, ATAC study demonstrated improvement of FACT-B TOI score in patients with previous chemotherapy than in those with no previous chemotherapy [12]. Our findings are in line with these two studies. The findings showed significant effects of all of these factors, except for mastectomy, on the changes in FACT-B TOI scores. Further exploratory analysis also showed that the TOI for the previous chemotherapy patient group >60 years of age without axillary lymph-node dissection was significantly improved from the baseline value. Additionally, effects of baseline score, follow-up, and prior chemotherapy on changes in FACT-B EWB subscale scores were significant, whereas the effects of age group, mastectomy, and axillary lymph-node dissection were not significant. We prospectively set the two subscales of FACT-B, i.e. EWB and SWB as secondary endpoints. We considered that EWB and SWB are more important to women compared with other subscales for the breast cancer, as they serve as an individual resource for dealing with illness and attuning to uncertainties related with chronic illness. It can also affect a woman’s self-esteem or may have psychological impact as a result of breast cancer. There were significant effects of baseline score, follow-up, previous chemotherapy, age group, and axillary lymph-node dissection on changes in FACT-B SWB subscale scores, but mastectomy had no significant effect. Although studies have analyzed the HRQoL outcomes following AI treatment, a definitive comprehensive conclusion on long-term HRQoL is still lacking in China. Several unresolved issues still prevent clinicians from designing an optimal endocrine therapy for postmenopausal women with EBC. Thus, the current study provides reinforcement to the previous findings and indicates improved long-term QoL with AI treatment. This will aid clinicians in optimizing treatment to yield effective healthcare outcomes.

Our results were different from those of ATAC and the Intergroup Exemestane Study (IES) substudy, which may be probably because of the following factors: (1) culture background: after treatment, American women with breast cancer were more likely to feel a loss of personal image, intimacy, and sex life than East Asian women [20, 21]; (2) study population: although the patients in ATAC, IES, and this study were menopausal women, there was no clear definition of “menopause” in the relevant published literature; therefore, it cannot be confirmed whether the definition was consistent with that in NCCN-Breast Cancer Version 2013 ( having previous bilateral oophorectomy; ≥60 years of age; <60 years of age having experienced amenorrhea for more than 12 months with no chemotherapy, tamoxifen, toremifene, or ovariectomy and follicle-stimulating hormone (FSH) and estradiol within menopausal range; <60 years of age having taken tamoxifen or toremifene, and FSH and plasma estradiol within menopausal range). Women ≤60 years of age accounted for only 33.7 % in the IES study, whereas women ≤60 years of age accounted for 65.0 % in this study.

Finally, the inherent limitations of this study should not be neglected. The study had neither a control or a standard group nor a blinding design. It is well known that a standard endocrine therapy for breast cancer would continue for at least 5 years; however, in the current study, all but 1 patient received the initial hormonal therapy at baseline and the follow-up was only for 2 years. Therefore, the effect of endocrine therapy on 3- to 5-year long-term QoL remains unknown. The aforementioned limitations indicate that this study only recognizes the significant improvements in the long-term QoL (6, 12, 18, and 24 months) of postmenopausal Chinese patients with HR+ EBC after starting treatment with AIs and does not provide a robust association between improvement of QoL scores ande treatment with AIs. Generally, observational studies have the benefit of assessing multiple aspects of lifestyle and better represent the results; however, potential limitation of selection bias and reporting of scores may have confounded the overall results in this study. We also did not assess the side effects of the treatment by aromatase inhibitors in this population. We had not systematically recorded the safety profile during the study execution and retrospective safety recall would have bias. Taken together, our study results showed the dynamic change of FACT-B, which will guide the physicians to communicate with the HR+ EBC patients about the QOL when receiving adjuvant AIs. Also, the improved QoL will increase patient compliance during the study treatment.

## Conclusions

The present study demonstrated significant improvements in the long-term QoL of postmenopausal patients with EBC at 6, 12, 18, and 24 months after starting treatment with AIs compared with baseline values. Based on results from the MIXED model (for data collection at 6, 12, and 18 months), we found significant effects of baseline score, follow-up, previous chemotherapy, age group, and axillary lymph-node dissection on changes in FACT-B TOI score and SWB scores, as well as significant effects of baseline score, follow-up, and previous chemotherapy on changes in FACT-B EWB scores. However, there were no significant effects of mastectomy on changes in TOI, SWB, or EWB scores. Further comparative work should be undertaken to strengthen the findings from this study.

## Abbreviations

AI: Aromatase inhibitor
ATAC: Arimidex, Tamoxifen, Alone or in Combination (trial)
BCS: Breast Cancer Subscale
CI: Confidence interval
EBC: Early-age breast cancer
ER+: Estrogen receptor-positive
ES: Endocrine subscale
EWB: Emotional well-being
FACT-B: Functional Assessment of Cancer Therapy-Breast
FACT-G: Functional Assessment of Cancer Therapy-General
FSH: Follicle-stimulating hormone
HR+: Hormone receptor-positive
HRQoL: Health-related QoL
ICF: Informed consent form
IES: Intergroup Exemestane Study
ITT: Intent to treat
LS: Least squares
NCCN: National Comprehensive Cancer Network
PR+: Progesterone receptor-positive
QoL: Quality of life
SD: Standard deviation
SFDA: State Food and Drug Administration
SWB: Social well-being
TOI: Trial outcome index
TNM: Cancer classification system based on tumour (T), lymph node (N) and metastase (M)
WHO: World Health Organization
